# Clinical implications of the family history in patients with lung cancer: a systematic review of the literature and a new cross-sectional/prospective study design (FAHIC: lung)

**DOI:** 10.1186/s12967-024-05538-4

**Published:** 2024-07-31

**Authors:** Fabrizio Citarella, Kazuki Takada, Priscilla Cascetta, Pierfilippo Crucitti, Roberta Petti, Bruno Vincenzi, Giuseppe Tonini, Francesco M. Venanzi, Alessandra Bulotta, Sara Oresti, Carlo Greco, Sara Ramella, Lucio Crinò, Angelo Delmonte, Roberto Ferrara, Massimo Di Maio, Fiorella Gurrieri, Alessio Cortellini

**Affiliations:** 1 Operative Research Unit of Medical Oncology, Fondazione Policlinico Universitario Campus Bio-Medico, Via Alvaro del Portillo, 200, 00128 Rome, Italy; 2 Department of Medicine and Surgery, Universitá Campus Bio-Medico Di Roma, Via Alvaro del Portillo, 21, 00128 Rome, Italy; 3 Department of Surgery, Saiseikai Fukuoka General Hospital, Fukuoka, Japan; 4 Department of Cancer Medicine, Gustave Roussy Cancer Campus, Villejuif, France; 5 Thoracic Surgery Department, Fondazione Policlinico Universitario Campus Bio-Medico, Via Alvaro del Portillo 200, 00128 Rome, Italy; 6 Operative Research Unit of Medical Genetics, Fondazione Policlinico Universitario Campus Bio-Medico, Via Alvaro del Portillo, 21, 00128 Rome, Italy; 7 Università Vita-Salute San Raffaele, Milan, Italy; 8 Department of Medical Oncology, IRCCS Ospedale San Raffaele, Milan, Italy; 9 Radiation Oncology, Fondazione Policlinico Universitario Campus Bio-Medico, Via Alvaro del Portillo, 200, 00128 Rome, Italy; 10 Thoracic Oncology Unit, IRCCS Istituto Romagnolo Per Lo Studio Dei Tumori (IRST) “Dino Amadori”, Meldola, Italy; 11 Department of Oncology, Medical Oncology 1U, AOU Città della Salute e della Scienza di Torino, University of Turin, 10126 Turin, Italy; 12 Department of Surgery and Cancer, Hammersmith Hospital Campus, Imperial College London, London, UK

**Keywords:** Lung cancer, NSCLC, Family history of cancer, Germline screening

## Abstract

Compared to other malignancies, few studies have investigated the role of family history of cancer (FHC) in patients with lung cancer, yielding largely heterogeneous results. We performed a systematic literature review in accordance with PRISMA guidelines, searching the PubMed and Scopus databases from their inception to November 25, 2023, to identify studies reporting on the role of FHC in patients with lung cancer. A total of 53 articles were included, most with a retrospective design and encompassing a variety of geographical areas and ethnicities.

Thirty studies (56.6%) assessed patients with non-small cell lung cancer (NSCLC), while 17 studies (32.1%) assessed patients with mixed histologies. Overall, the rates of FHC ranged from 8.3 to 68.9%, and the rates of family history of lung cancer ranged from 2 to 46.8%. Twenty-seven studies investigated FHC as a potential risk factor for lung cancer, with more than half reporting an increased risk for subjects with FHC. Five studies reported on the potential role of FHC in determining clinical outcomes, and twelve studies examined the relationship between FHC and germline mutations. Notably, only one study reported a significantly increased rate of germline mutations, including *ATM*, *BRCA2*, and *TP53*, for patients with a family history of lung cancer compared to those without, but both groups had a low prevalence of mutations (< 1%).

The FAHIC—Lung (NCT06196424) is the first cross-sectional/prospective study specifically developed to identify FHC patterns and within-family clusters of other risk factors, including smoking, to guide patients with NSCLC to systematic genetic counseling. Acknowledging the largely heterogeneous results of our systematic review and considering the clinical implications of detecting pathogenic germline variants (PGVs), the FAHIC-lung study aims to identify patients potentially enriched with PGVs/likely PGVs to direct them to germline screening outside of the research setting.

**Supplementary Information:**

The online version contains supplementary material available at 10.1186/s12967-024-05538-4.

## Introduction

Familial aggregation and inherited predisposition have been increasingly investigated in multiple cancer types. In breast, ovarian, prostate, and colorectal malignancies, international guidelines recommend genetic counselling in patients showing risk criteria for syndromes of inherited susceptibility to cancer, as aggregations with other malignancies have been widely described within families of these patient populations [1–3].

With a predicted number of death of about 160 000 cases in 2023 in Europe and 127 070 in US [4, 5], Non-Small Cell Lung Cancer (NSCLC) still remains a leading cause of cancer death worldwide. A positive smoking history represents the main risk factor [6], while environmental factors such as exposure to radon, asbestosis and air pollution have been linked to lung cancer among never smokers [7–9].

Few studies have investigated the impact of a positive family history of cancer (FHC) in patients with NSCLC, describing the malignancies that can occur among relatives of patients with NSCLC, while only few and rare genetic syndromes associated with inherited germline genetic mutations, such as the Li-Fraumeni, have been directly linked to lung cancer risk [10]. Most of the studies did not provide information on the potential within-family clusters of other risk factors, including exposure to tobacco smoking, environmental carcinogens, and other geographical/epidemiological factors. Additionally, retrospective approaches to this topic are heavily impacted by recall bias and misclassification [11, 12].

To underline the importance and potential clinical implications of investigating family history of cancer (FHC) in patients with non-small cell lung cancer (NSCLC), a recent retrospective study conducted in a cohort of 7.788 patients with NSCLC, who underwent commercially available germline genetic testing and reported an FHC of 71%, found that pathogenic germline variants (PGVs) or likely PGVs were present in 14.9% of the cases. Additionally, 2.9% of the cases carried a single PGV in a gene associated with autosomal recessive inheritance. Among positive patients, 61.3% carried a PGV/likely PGV in DNA damage and response (DDR) genes, and 95.1% of them harbored a PGV in genes with potential clinical implications, including *BRCA2* (2.8%), *CHEK2* (2.1%), *ATM* (1.9%), *TP53* (1.3%), *BRCA1* (1.2%), and *EGFR* (1.0%) [13].

In this manuscript, we present the results of a systematic review of the available evidence on the role of FHC in patients with lung cancer, and the design of the FAHIC-lung study (NCT06196424), a cross-sectional study that aims to prospectively describe the FHC and the potential within-family distribution of smoking and other risk factors, to identify patients more likely to be carriers of PGVs or likely PGVs.

## Systematic review—methods

### Literature search strategy and study selection criteria

This systematic review was conducted in accordance with the Preferred Reporting Items for Systematic Reviews and Meta-Analyses (PRISMA) guidelines. We searched the PubMed and Scopus databases from their inception date to November 25, 2023, to identify potentially relevant articles. The search terms were “non-small cell lung cancer or NSCLC,” “family history,” “lung cancer,” and “risk.”

The inclusion criteria for the study selection were as follows: (1) patients diagnosed with NSCLC of any stage; (2) available information on the family history of cancer for the included population (e.g., prevalence and type of family history). The exclusion criteria were as follows: (1) lack of information on the family history of cancer; (2) studies not published in English; and (3) case reports.

As this study was a systematic review, ethical approval and informed consent were not required. The study protocol was registered in PROSPERO, an international prospective register of systematic reviews funded by the National Institute for Health Research (NIHR), with the registration code CRD4202450742 (available at: https://www.crd.york.ac.uk/prospero/display_record.php?ID=CRD42024507422).

### Data extraction and data synthesis

Two authors (F.C. and K.T.) performed the literature search and evaluated the eligibility of studies using the PICO (patients, interventions, comparison, and outcome) framework following the PRISMA criteria. Assuming a certain heterogeneity in the results, we adopted a textual narrative synthesis approach to summarize the included publications [14]. In view of that, we did not establish specific criteria for data synthesis (e.g., the minimum number of studies or level of consistency required for synthesis).

F.C. and T.K. independently reviewed and extracted data from the published papers, including first author, journal name, and year of publication. The prevalence (as a rate) of family history of cancer was summarized in a master table, along with the type of family history collected (e.g., lung-cancer specific vs. family history of any malignancy), study design, study population characteristics, smoking status of study participants and screened relatives (if available), primary tumor type (e.g., NSCLC, small cell lung cancer [SCLC], or others), number of patients included, and disease stage (e.g., early stage vs. advanced stage, if available). Study characteristics, context, and findings were summarized, and similarities/differences across studies were described in detail. Disagreements between the two authors (F.C. and K.T.) were discussed and resolved with a third independent author (A.C.).

## Systematic review—results

**Fig. 1:**
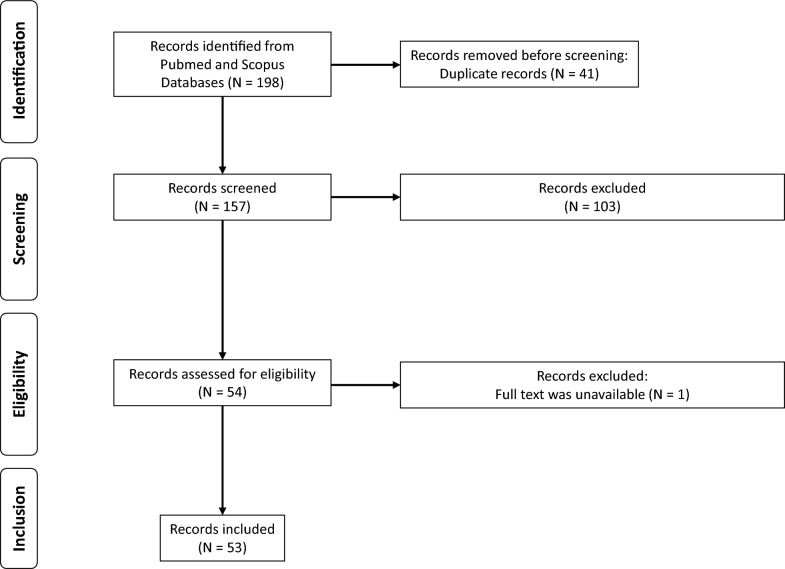
Flow diagram of the studies selection process according to the PRISMA guidelines

We identified a total of 198 potentially relevant articles from the PubMed and Scopus online databases through an initial search strategy. After excluding 41 duplicate articles, we screened and reviewed the titles and abstracts of 157 articles, resulting in 54 being assessed for eligibility. Finally, a total of 53 articles were included in this systematic review. The flow diagram of the study selection process is shown in Fig. 1 while the whole search strategy with publications assessed at each step (identification, screening, eligibility and inclusion) is available as supplementary material (search strategy).

Overall, the vast majority of the studies had a retrospective design, with most of them being case–control or observational retrospective studies, with only one cross-sectional study [15] and one prospective study [16]. Study populations encompassed a variety of geographical areas/ethnicities, with 23 studies (43.4%) enrolling Asian patients, 13 studies (24.5%) enrolling patients with multiple ethnicities (all with a majority of white patients), 11 studies (20.7%) including non-specified ethnicities, and six studies (11.3%) including other populations. Even the included histology types showed heterogeneity, with 30 studies (56.6%) assessing patients with NSCLC, 17 studies (32.1%) assessing patients with a mixed type of lung cancer including small cell lung cancer (SCLC), four studies (7.5%) assessing other/unspecified types of lung cancer, one study (1.9%) assessing patients with adenocarcinoma, and one study (1.9%) assessing patients with *EGFR*-positive adenocarcinoma only.

FHC was collected through questionnaires in only three studies [17–19], while none of them used ad-hoc questionnaires specifically developed to collect FHC and the within-family distribution of other risk factors, including smoking. Twenty-five studies (47.2%) assessed family history (FH) by collecting all malignancies reported among relatives, 21 studies (39.6%) assessed FH of lung cancer, three studies (5.7%) assessed FHC and FH of lung cancer separately, three studies (5.7%) assessed FH of smoking-related and smoking-unrelated cancers, and two studies (3.8%) assessed FH of pre-specified types of cancer. The degree of relatedness ranged from first to second degree, although it was not reported for the majority of the included studies. One study reported on the smoking status among the relatives of study participants [20] and one study included the assessment of environmental factors (coal exposure) among the risk factors for lung cancer [21].

Overall, the rate of FHC in patients with lung cancer ranged from 8.3 [22] to 68.9% [20], while the rates of FH of lung cancer from 2 [23] to 46.8% [21]. Some studies enrolled cohorts of patients potentially enriched for FHC, such as 11 studies which assessed female patients only reporting FHC ranging from 7.7 [24] to 59.4% [25] and FH of lung cancer ranging from 6.2 [26] to 28% [27], four studies which specifically assessed never/light smoker patients only, reporting FHC ranging from 29.1 [28] to 68.9 [20], two studies assessing patients with small aggressive NSCLC, one study assessing male patients only, one study assessing smokers specifically, and one study assessing patients aged ≤ 45 years. A synoptic table with organization of results is available as supplementary file 1.

### Studies investigating FHC as a risk factor for lung cancer

Overall, 27 studies investigated FHC as a potential risk factor for lung cancer (Table 1) [16, 17, 20, 23–27, 29–47]. Six out of 11 studies (54.5%) that investigated the role of FHC as a whole or in pre-specified type of cancers reported an increased risk of developing lung cancer for patients with FHC, while 11 out of 16 studies (68.7%) that investigated the role of FH of lung cancer reported a significant association.

**Table 1:** summary of the included studies reporting on the potential role of FHC as a risk factor for lung cancer

Study	Design	Country/Race	Study Population	Histology	Type of FHC	Prevalence of FHC among cases	Smoking status details (cases)	Main findings	Additional findings
Tsugane et al Jpn J Clin Oncol (1987)	Case–control study	Japan/Asian	185 patients with lung cancer diagnosis between 30 and 49 years; 134 matched controls	NSCLC, SCLC and others	Any cancer (up to second-degree relatives)	FHC 36% and 32% for male and female patients with adenocarcinoma respectively FH of lung cancer of 6% and 2% for male and female patients with adenocarcinoma respectively FHC of 30% and FH of lung cancer of 5% among patients with squamous cell carcinoma	Adenocarcinoma histology: 75% male patients were ever smokers,15% of female patients were ever smokers > 90% of patients with other tumors were ever smokers	FHC was not associated with diagnosis of early onset lung cancer	–
Osann Cancer Res (1991)	Case–control study	USA (86% White, 11% black, and 3% Asian)	217 females diagnosed with lung cancer; 217 matched controls	NSCLC, SCLC and others	Any cancer categorized as smoking related and smoking unrelated tumors (first-degree relatives)	FHC of 22.6% and 45.2% among never and ever smokers. FH of lung cancer of 3.2% and 9.0% among never and ever smokers	84.8% of ever smokers	FHC was significantly associated with diagnosis of lung cancer (OR 1.8, 95% CI 1.0–3.2)	Stronger association in women aged ≤ 55 (OR 3.8, 95% CI 0.9– 16.2) than those aged > 55 years (OR 1.4, 95% CI 0.7–2.6) Smoking and FHC showed synergistic effect in increasing risk of lung cancer
Gorlova et al Int J Cancer (2006)	Case–control study	USA/White (82.5%), Hispanic (8.9%), Black (8.6%)	280 cases of lung cancer in never/light smokers (< 100 cigarettes in lifetime); 242 unmatched healthy controls	NSCLC, SCLC and others	Any cancer, any smoking-related cancer (lung, head and neck, kidney, bladder and pancreatic), lung cancer, lung cancer in at least 1 never-smoker relative, any cancer detected before 50 years	FHC of 68.9%, FH of smoking-related cancer of 20.8%, FH of lung cancer of 13.2%, FH of lung cancer in at least one never smokers of 2.3%; FH of any cancer among relatives aged < 50 years 31.5%	All cases were never smokers (< 100 cigarettes in lifetime)	No significant association between overall FHC, FH of smoking-related cancers, and FH of lung cancer, and diagnosis of lung cancer	FH of any caner among relatives aged < 50 years was associated with the diagnosis of lung cancer (OR 1.87, 95% CI 1.13–3.1)
Chen et al Cancer (2007)	Case–control study	Taiwan/Asian	826 female patients with lung cancer and 531 unmatched heathy controls	NSCLC, SCLC and others	Breast, ovarian, cervical, and endometrial cancers (first-degree relatives)	FHC (breast, ovarian, cervical and endometrial) 7.7%	7.1% of ever smokers 69.5% of patients were exposed to passive smoking	No difference in FHC between cases and controls	Association between Hormone Replacement Therapy (HRT) and reduced risk of lung cancer among patients with negative FHC (OR 0.69, 95% CI 0.51–0.95)
Schwartz et al J Clin Oncol (2007)	Case–control study	USA/White (77%), Black (23%)	488 women with NSCLC; 498 matched controls	NSCLC	Lung cancer (first-degree relatives)	FH of lung cancer of 25%	8% of never smokers, 32% of former smokers, 60% of current smokers	FH of lung cancer was significantly associated with diagnosis of NSCLC (p < 0.001)	–
Tammemagi et al Cancer Epidemiol Biomarkers Prev (2007)	Observational, retrospective Case-case study	Canada/White (87.5%), Black (4.2%), Hispanic (2.1%), Asian (6.3%)	48 cases with Small Aggressive NSCLC (SA-NSCLC) and 329 controls enrolled in the Prostate, Lung, Colorectal and Ovarian Cancer Screening Trial (PLCO)	NSCLC	Lung cancer (first-degree relatives)	FH of lung cancer of 26.1%	10.4% of never smokers, 52.1% of former smokers, 37.5% of current smokers	FH of lung cancer was not associated with diagnosis of SA-NSCLC	In patients with FH of lung cancer diagnosis of SA-NSCLC was significantly more frequent among females
Cassidy et al Eur J Cancer (2009)	Case–control study	Europe/not available	733 surgically resected patients; 1312 matched controls	NSCLC	Any cancer, lung cancer, smoking-related cancers, and gastric cancer (first-degree relatives)	FH of any cancer 51.5%, FH of lung cancer 18.9%	7.1% of never smokers, 75.4% of former smokers, 17.5% of current smokers	FH for any cancer significantly associated with diagnosis of risk of NSCLC (OR 1.16, 95%: 1.02–1.33)	FH of lung cancer, FH of smoking-related cancers, and FH of non-smoking related cancers not associated with diagnosis of NSCLC Significant association between FH of gastric cancer and diagnosis of NSCLC, between FH of gastric cancer and late-onset NSCLC (≥ 55 years), and between FH of lung cancer and diagnosis of early-onset NSCLC (< 55 years)
Cote et al Carcinogenesis (2009)	Case–control study	USA/White (48.9%), Black (48.7%)	504 female patients with NSCLC; 527 matched controls	NSCLC	Lung cancer (first-degree relatives)	FH of lung cancer of 25.8%	7.9% of never smokers, 31.6% of former-smokers, 60.5% of current smokers	FH of lung cancer associated with diagnosis of NSCLC in both white and black patients (p < 0.01)	FH of lung cancer was among the factors in the most parsimonious model able to predict risk of NSCLC in white ever-smoker women together with age at diagnosis, history of chronic obstructive lung disease, pack-years of cigarette smoking, BMI, XRCC1 A/A genotypes, GSTM1 null and COMT A/G or G/G phenotype
Hong et al J Prev Med Public Health (2011)	Case–control study	Korea/Asian	406 patients with NSCLC; 428 unmatched controls	NSCLC	Any cancer (unspecified degree and tumors among relatives)	FHC of 20.4% (cases)	85.2% of ever smoker	FHC was not associated with diagnosis of NSCLC	In patients without FHC, the miR-196a2 CT/TT germline genotype was associated with diagnosis of NSCLC
Schwartz et al Carcinogenesis (2011)	Case–control study	USA/Black (African-Americans)	837 cases with lung cancer; 975 matched controls	NSCLC, SCLC and others	Lung cancer (unspecified degree among relatives)	FH of lung cancer of 22%	8.2% of never smokers, 91.8% of ever smokers	FH of lung cancer was significantly associated with diagnosis of lung cancer (p < 0.001)	Excess European ancestry on chromosome 1 at rs6587361 for NSCLC. Excess African ancestry on chromosome 3 at rs181696 among ever smokers with NSCLC
Sin et al J Clin Oncol (2013)	Retrospective study with exploratory and validation cohorts	Canada/White (97.6%), Asian (0.9%), Black (0.6%)	2,485 patients from the Pan-Can Study 61 Cases and 122 matched controls from the CARET study	NSCLC	Lung cancer (unspecified degree among relatives)	FH of lung cancer 5.37%	All patients reported a history of smoking	FH and risk of lung cancer were not associated with diagnosis of NSCLC in the Pan-Can cohort	Pro–Surfactant Protein B (SFTPB—log transformed) associated with diagnosis of lung cancer No association between FH of lung cancer and SFTPB
Xu et al PLoS One (2013)	Case–control study	China/Asian	1017 male patients with lung cancer; 1017 matched healthy controls 242 cases assessed for clinical outcomes	NSCLC, SCLC and others	Any cancer (unspecified degree and tumors among relatives)	FHC of 12.8% (cases)	14.8% of never smokers, 36.4% of former smokers 48.8% of current smokers	FHC was significantly associated with the diagnosis of lung cancer	Germline rs1564483GA, AA and GA + AA BCL2 SNPs were associated with decreased risk of lung cancer among patients with not FHC
He et al J Hum Genet (2013)	Case–control study	China/Asian	507 patients with NSCLC; 662 unmatched controls	NSCLC	Lung cancer (unspecified degree among relatives)	FH of lung cancer of 21.7% (cases)	36.9% of never smokers, 43.1% of ever smokers	FH of lung cancer was associated with the diagnosis of NSCLC (OR 1.47, 95%CI: 1.09–1.98) FH of lung cancer was associated with the diagnosis of NSCLC even among smokers (OR 1.94, 95%CI: 1.21–3.12)	Several XRCC3 and XRCC4 germ-line SNPs were associated with the diagnosis of NSCLC Some XRCC3 and XRCC4 haplotypes and diplotypes were associated with diagnosis of NSCLC, with synergistic effect for increased risk with FH of lung cancer
Yilmaz et al Asian Pac J Cancer Prev (2014)	Case–control study	Turkey/not available	100 patients with lung cancer; 100 matched healthy controls	NSCLC, SCLC and others	Any cancer (unspecified degree and tumors among relatives)	FHC of 23%	90% of smokers	FHC was significantly associated with diagnosis of lung cancer diagnosis (p = 0.001)	No association between MTHFR C677T polymorphism and diagnosis of lung cancer
Pathak et al J Cancer Ther Res (2014)	Case–control study	USA/White (83%), Black (17%)	453 female patients with NSCLC; 478 matched controls	NSCLC	Lung cancer (unspecified degree and tumors among relatives)	FH of lung cancer of 24% among white participants and 28.7% among Black participants	90.3% and 91.5% of ever smokers among White and African American, respectively	FH of lung cancer was associated with diagnosis of lung cancer in both whites and Black participants (p < 0.01)	Germline single nucleotide polymorphisms (SNP) APAF-1 rs1007573 and CD40 rs1535045 were associated with lung cancer in white participants, while SNP TP63 rs6790167 was associated with lung cancer in black participants
Tian et al Cell Biochem Biophys (2014)	Case–control study	China/Asian	391 patients with NSCLC; 663 matched controls	NSCLC	Any cancer (unspecified degree and tumors among relatives)	FHC of 27.4% (cases)	25.8% of ever smokers	FHC was associated with the diagnosis of NSCLC	Significant enrichment of germline NQO1 C609T TT SNP compared to CC among cases Germline NQO1 C609T TT SNP was associated with diagnosis of NSCLC after adjustment for FHC
Li et al Int J Clin Exp Med (2016)	Case–control study	China/Asian	420 patients with lung cancer aged ≤ 45 years; 1673 matched controls	NSCLC, SCLC and others (cases)	Lung cancer (first-, second-degree relatives and collateral relatives)	FH of lung cancer of 7.3%	27.6% of never smokers, 72.4% of ever smokers	FH of lung cancer was associated with diagnosis of lung cancer risk (p = 0.03)	FH of lung cancer among first- and second-degree relatives more strongly associated with diagnosis of lung cancer than FH of lung cancer among collateral relatives
White and Horvitz (2017)	Case–control study, exploratory	USA/not available	5443 web-identified potential cases and remaining 4,813,985 web users as controls	Likely diagnosis of lung cancer	Lung cancer (unspecified degree among relatives)	NA	NA	FHC associated with likely diagnosis of lung cancer (RR 7.548, 95% CI 3.9–14.4)	–
Tammemagi et al Lancet Oncol (2017)	Prospective, single arm study	Canada/White (97%), Black (3%)	164 patients with lung cancer among 2537 ever-smoker enrolled subjects between 50 and 75 years without history of cancer at enrollment	NSCLC, SCLC and others	Lung cancer (first-degree relatives)	FH of lung cancer 39%	All patients reported a history of smoking	No significant association between FH of lung cancer and diagnosis of lung cancer within the study population	-
Warkentin et al JNCI Cancer Spectr (2018)	Observational, retrospective. Case-case study	USA/White (96.9%)	64 patients with SA-NSCLC and 206 non-SA-NSCLC cases enrolled in the National Cancer Institute’s National Lung Screening Trial	NSCLC	Lung cancer (first-degree relatives)	FH of lung cancer 31.8%	All patients reported a history of smoking	FH of lung cancer was not associated with diagnosis of SA-NSCLC	FH of lung cancer associated with SA-NSCLC among female patients
Brown et al Cancer Epidemiol Biomarkers Prev (2019)	Case–control study	USA/White (93.5%), Black (5.3%)	262 cases with lung cancer; 528 matched controls	NSCLC	Lung cancer (unspecified degree among relatives)	FH of lung cancer 22.9%	All patients reported a history of smoking	No significant association between FH of lung cancer and diagnosis of lung cancer within the study population	–
Titan et al J Thorac Cardiovasc Surg (2020)	Observational, retrospective	USA/ White (88.6%)	75,587 female patients aged from 50 to 74 years enrolled in the Prostate, Lung, Colorectal, and Ovarian Cancer Screening Trial (PLCO)	1147 patients with NSCLC	Any cancer and Lung cancer (unspecified degree and tumors among relatives)	FHC of 59.4% and FH of lung cancer of 11.4% (whole study cohort)	NA	FH of lung cancer was associated with the risk of developing NSCLC over time (HR 1.79, 95%CI: 1.56–2.06)	FH of lung cancer is associated with the risk of developing NSCLC independently of hormone replacement therapy
Jin et al Int J Cancer (2021)	Case–control study	Japan, China, USA, Singapore, Malaysia and others/Asian	Pooled analysis of the International Lung Cancer Consortium (ILCCO) including 264 women with lung cancer and 5342 matched controls	NSCLC, SCLC and others	Lung cancer (first-degree relatives)	FH of lung cancer 6.2%	65.3% of never smokers, 10.5% of former smokers, 23.7% of current smokers	FH of lung cancer was significantly associated with diagnosis of lung cancer (p < 0.01)	–
Lancheros et al Nutrients (2022)	Case–control study	Spain/Caucasian	204 cases of NSCLC; 408 unmatched controls	NSCLC	Any cancer (unspecified degree and tumors among relatives)	FHC of 49.5% (cases)	13.24% of never smokers, 39.71% of former smokers, 47.96% of current smokers	FHC was associated with diagnosis of NSCLC (OR 15.2, 95%CI: 9.55–25.2)	The Vitamin D Receptor (VDR) BsmI rs1544410-AA germline SNP was associated with lower risk of NSCLC after adjusting for FHC and smoking
Albano et al Cancer Epidemiol (2023)	Observational, retrospective	USA/not available	16,056 never smoker patients with 579 cases of lung cancer	Lung cancer (not specified)	Lung cancer (unspecified degree among relatives)	FH of lung cancer 28.7%	NA	FH of lung cancer was associated with diagnosis of lung cancer (OR 1.87, 95% CI 1.55–2–26)	–
Rifkin et al. Clin Lung Cancer (2023)	Case–control study	United Kingdom/Withes (94.7%)	2050 patients with lung cancer/198533 controls	Lung cancer (not specified)	Lung cancer (unspecified degree among relatives)	FH of lung cancer of 21.0%	20% of never smokers, 46.6% of former smokers, 33.4% of current smokers	FH of lung cancer was significantly associated with diagnosis of lung cancer	FH of lung cancer (whole cohort including cases and controls) was significantly associated to germline mutations in 3 significant genes (ATM, BRCA2, TP53)
Liu et al Nutrients (2023)	Observational, retrospective	China/Asian	1283 patients with NSCLC and 215 patients with benign lung nodules	NSCLC	Any cancer (unspecified degree and tumors among relatives)	FHC 20.7%	62.9% of never smokers, 14.2% of former-smokers, 22.9% of current smokers	FHC was not associated with diagnosis of NSCLC	FHC was associated with diagnosis of intrapulmonary metastasis among patients with NSCLC at univariable analysis

NSCLC: non-small cell lung cancer; SCLC: small cell lung cancer; OR: odds ratio; RR: relative risk; HR: hazard ratio; 95%CI 95% confidence intervals; SNPs: single nucleotide polymorphisms

One study reported a more pronounced increased risk for women aged ≤ 45 years and a synergistic effect of smoking and FHC in increasing the risk of lung cancer [29], while another study reported that FH of lung cancer was specifically associated with an increased risk of early on set lung cancer (< 55 years old) [17]. One study that failed to demonstrate an association between FHC and lung cancer diagnosis, reported a significant effect for patients in whom at least one relative with cancer was diagnosed < 50 years of age [20], while one study that failed to demonstrate an association between FH of lung cancer and lung cancer risk, reported a significant effect for female patients only [42].

One study confirmed that FH of lung cancer was associated with risk of lung cancer in both the whole study population and among smokers [37], while another study reported that FH of lung cancer was more strongly associated with lung cancer risk in case of first/second degree of relatedness compared to collateral relatives [40].

### Studies investigating the potential impact of FHC on clinical outcomes.

Five studies reported on the potential role of FHC in determining clinical outcomes (Table 2) [19, 28, 48–52]. One study reported no association between FH of lung cancer and outcomes [48], two studies reported a differential effect for FHC and FH of lung cancer [28, 49] and one study reported a decreased risk of death for patients with FHC [50]. Similarly, one study reported improving outcomes from PD-1 immunotherapy with increasing burden of FHC [52].

**Table 2:** summary of the included studies reporting on the potential implication of FHC on clinical outcomes

Study	Design	Country/Race	Study Population	Histology	Type of FHC	Prevalence of FHC among cases	Smoking status details (cases)	Main findings	Additional findings
Yang et al Ann Clin Lab Sci (2008)	Observational, retrospective	USA/not available	133 patients with resected stage I-IIIA NSCLC	NSCLC	Lung cancer (first-degree relatives)	FH of lung cancer of 43%	8% of never smokers, 58% of former smokers, 35% of current smokers	FH of lung cancer was not associated with relative risk of death	FH of lung cancer was not associated with hHpr1/p84/Thoc1 expression on tumor samples
Li et al Fam Cancer (2011)	Observational, restrospective	China/Asian	539 patients with FHC, including 233 patients with FH of lung cancer among a cohort of 4491 patients with NSCLC	NSCLC	Any cancer (first-degree and second-degree relatives)	FHC of 12%, FH of lung cancer of 5.2%	Among patients with no FHC 66.2% pf ever smokers Among patients with FHC 60.1% of ever smokers	FHC was significantly associated with decreased response to chemotherapy (p = 0.024) FH of lung cancer was associated with improved survival among early-stage NSCLC (p = 0.015)	FHC was significantly associated with adenocarcinoma histology, advanced stage disease, non-smoking status, younger age at diagnosis and female gender FH of lung cancer was significantly associated with younger age at diagnosis, adenocarcinoma histology, advanced stage disease
Li et al Life Science Journal (2013)	Observational, prospective	China/Asian	60 patients undergoing resection for NSCLC	NSCLC	Any cancer (unspecified degree and tumors among relatives)	FHC of 20%	61.7% of ever smokers	FHC was significantly associated with reduced relative risk of death (RR 0.52, 95%CI: 0.16–0.92)	The expression of ERCC1 mRNA was significantly associated with shorter survival
Su et al Cell Physiol Biochem (2015)	Observational, retrospective	China/Asian	610 patients with NSCLC	NSCLC	Any cancer (unspecified degree and tumors among relatives)	FHC of 17.5%	29.0% of never smokers, 23.28% of former smokers, 47.7% of current smokers	FHC was not associated with survival	Germline *hOGG1* (rs1052133 C > G) SNP was associated with shorter survival among patients without FHC The hOGG1 G allele correlated with shorter survival among patients without FHC (HR 1.60, 95%CI: 1.04 – 2.45)
Isla et al Anticancer Res (2016)	Case–control study	Spain/not available	876 female patients with FHC and 886 female patients without FHC	NSCLC and SCLC	Any cancer (first-degree and second-degree relatives)	FHC of 42.5% among the whole cohort; 43.5% among patients with SCLC; 42.4% among patients with NSCLC	Among patients without FHC: 43% of never smokers, 15.7% of former smokers, 40.5% of current smokers Among patients with FHC: 36.4% of never smokers, 15.6% former smokers, 47.2 current smokers	FHC was not associated with tumor type (NSCLC vs SCLC) Positive smoking personal history was significantly associated with FHC (p = 0.036)	Positive FHC was associated with shorter OS (23 vs 25.3 months, p = 0.029)
Lee et al Lung Cancer (2019)	Observational, retrospective	Korea/Asian	604 female patients who never smoked with resected lung adenocarcinoma	Adenocarcinoma	Any cancer, categorized as pulmonary and non-pulmonary cancer (first degree relatives)	FHC of 29.1%, FH of lung cancer 7.3%	–	FH of non-pulmonary cancer was significantly associated with higher risk of recurrence (HR 1.9, 95%CI: 1.40–2.56) and death (HR 1.67, 95%CI: 1.18–2.37), compared with those with no FHC FH of pulmonary cancer was not associated with recurrence and/or death	FH of non-pulmonary cancer was associated with younger age at diagnosis FH of pulmonary cancer was associated with an increased rate of EGFR mutation FH of non-pulmonary cancer was associated with ALK/ROS-1/RET fusion status No association between FH and KRAS status Non-significant increase of CDH1 germline mutations in patients with non-pulmonary FH
Cortellini et al J Hematol Oncol (2022)	Case control study	Italy/not specified	723 patients with NSCLC treated with pembrolizumab (cases); 652 patients with NSCLC treated with chemotherapy (controls)	NSCLC	Any cancer in lineal line (descendants or ascendants) and collateral line (non-descendants/ascendants, relatives)	FHC of 37.5% (cases) 49.3% (controls)	Pembrolizumab cohort: 12.4% of never smokers and 87.6% of ever smokers Chemotherapy cohort: 12.6% of never smokers and 87.4% of ever smokers	High burden of FHC associated with improved outcomes compared to low burden/negative FHC among patients treated with pembrolizumab only	FHC was not associated with tumor mutational burden, PD-L1 status and somatic DNA damage and response genes status

NSCLC: non-small cell lung cancer; SCLC: small cell lung cancer; RR: relative risk; HR: hazard ratio; 95%CI 95% confidence intervals

### Studies investigating associations between FHC and germline mutations.

Overall, 12 studies reported on the potential relationship between FHC and germline mutations (Table 3) [33, 36, 37, 39, 44, 46, 51, 53–57]. Two studies did not show an enrichment of the germline mutations/polymorphisms of interest in patients with FHC [53, 55], while three studies suggested a potential enrichment [46, 54, 57], with only one of them specifically reporting an increased rate of germline mutations including *ATM, BRCA2* and* TP53* for patients with family history of lung cancer compared to those with no FH [46]. Two studies reported a significant effect of the germline status in increasing the risk of lung cancer among patients with no FHC [33, 36], while in three other studies the effect was independent of FHC [39, 44, 46]. One study showed a synergistic effect in increasing the risk of lung cancer of *XRCC3/XRCC4* variants and FHC [37]. Two studies investigated the potential impact of germline polymorphisms on clinical outcomes, one showing an association between *hOGG1* single nucleotide polymorphisms and worse survival specifically in patients without FHC [51], the other showing multifaceted effects of germline NOTCH4 polymorphisms depending on the FHC status [56].

**Table 3:** summary of the included studies reporting on the potential association between FHC and germline mutations

Study	Design	Country/Race	Study Population	Histology	Type of FHC	Prevalence of FHC among cases	Smoking status details (cases)	Main findings	Additional findings
Tefre et al Br J Cancer (1990)	Case–control study	Norway/not available	83 patients with lung cancer; 129 unmatched healthy controls	SCLC and NSCLC	Any cancer (unspecified degree and tumors among relatives)	Not available	4.8% of never smokers	FH of cancer was not associated with germline EcoR1 polymorphism of L-myc	–
Hong et al J Prev Med Public Health (2011)	Case–control study	Korea/Asian	406 patients with NSCLC; 428 unmatched controls	NSCLC	Any cancer (unspecified degree and tumors among relatives)	FHC of 20.4% (cases)	85.2% of ever smoker	FHC was not associated with diagnosis of NSCLC	In patients without FHC, the miR-196a2 CT/TT germline genotype was associated with diagnosis of NSCLC
Xu et al PLoS One (2013)	Case–control study	China/Asian	1017 male patients with lung cancer; 1017 matched healthy controls	NSCLC, SCLC and others	Any cancer (unspecified degree and tumors among relatives)	FHC of 12.8% (cases)	14.8% of never smokers, 36.4% of former smokers 48.8% of current smokers	FHC was significantly associated with the diagnosis of lung cancer	Germline rs1564483GA, AA and GA + AA BCL2 SNPs were associated with decreased risk of lung cancer among patients with not FHC
He et al J Hum Genet (2013)	Case–control study	China/Asian	507 patients with NSCLC; 662 unmatched controls	NSCLC	Lung cancer (unspecified degree among relatives)	FH of lung cancer of 21.7% (cases)	36.9% of never smokers, 43.1% of ever smokers	FH of lung cancer was associated with the diagnosis of NSCLC (OR 1.47, 95%CI: 1.09–1.98) FH of lung cancer was associated with the diagnosis of NSCLC even among smokers (OR 1.94, 95%CI: 1.21–3.12)	Several XRCC3 and XRCC4 germ-line SNPs were associated with the diagnosis of NSCLC Some XRCC3 and XRCC4 haplotypes and diplotypes were associated with diagnosis of NSCLC, with synergistic effect for increased risk with FH of lung cancer
Tian et al Cell Biochem Biophys (2014)	Case–control study	China/Asian	391 patients with NSCLC; 663 matched controls	NSCLC	Any cancer (unspecified degree and tumors among relatives)	FHC of 27.4% (cases)	25.8% of ever smokers	FHC was associated with the diagnosis of NSCLC	Significant enrichment of germline NQO1 C609T TT SNP compared to CC among cases Germline NQO1 C609T TT SNP was associated with diagnosis of NSCLC after adjustment for FHC
Su et al Cell Physiol Biochem (2015)	Observational, retrospective	China/Asian	610 patients with NSCLC	NSCLC	Any cancer (unspecified degree and tumors among relatives)	FHC of 17.5%	29.0% of never smokers, 23.28% of former smokers, 47.7% of current smokers	FHC was not associated with survival	Germline *hOGG1* (rs1052133 C > G) SNP was associated with shorter survival among patients without FHC The hOGG1 G allele correlated with shorter survival among patients without FHC (HR 1.60, 95%CI: 1.04—2.45)
Javid et al Clin Transl Oncol (2015)	Case–control study	India/not available	155 patients with NSCLC; 155 matched controls	NSCLC	Any cancer (unspecified degree and tumors among relatives)	FHC of 12.2% (cases)	28.4% of never smokers, 71.6% of ever smokers	Polymorphism in the inhibitory P2 promoter region of anti-apoptotic BCL-2 genes was associated with diagnosis of NSCLC (-938CC/AC)	Patients with FHC enriched in BCL92 (-938AA) genotype
Liu et al J Thorac Oncol (2016)	Case–control study	USA/White	54 patients with familial lung cancer and 48 patients with sporadic lung cancer	NSCLC, SCLC and others	Lung cancer (in at least 3 first-degree relatives)	–	14.8% of never smokers, 85.2% of ever smokers	Patients with FH of lung cancer were younger, with higher prevalence of female and never smokers than those with sporadic lung cancer,	30 germline variants of interest found in 33.3% of patients with FH and 29.2% of patients with no FH Patients with FH of lung cancer were not significantly enriched of deleterious mutations found in the whole study population [heterozygous c.2086C > T in the coiled-coil domain-containing 147 gene (CCD147), and two SNV p.Val26Met and p.Met563Thr, of the dopamine beta-hydroxylase gene (DBH)]
Xu et al Cancer Manag Res (2019)	Observational, retrospective	China/Asian	987 patients with NSCLC	NSCLC	Any cancer (unspecified degree and tumors among relatives)	FHC of 20.2%	62.4% of never smokers, 37.6% of ever smokers	No association between FHC and survival	Germline rs915894 AC/CC genotype of the *NOTCH4* gene was associated with decreased risk of death, with a greater effect among patients with FHC Patients with rs915894 AA genotype of the *NOTCH4* gene and positive FHC showed increased risk of death
Liu et al Transl Lung Cancer Res (2020)	Retrospective study	China/Asian	1026 patients with NSCLC	NSCLC	Lung cancer (in at least one first-degree relatives)	FH of lung cancer of 26.8%	43.1% of never smokers, 56.9% of ever smokers	Germline mutations categorized as pathogenic, likely pathogenic or non-pathogenic group	Patients with FH of lung cancer were significantly enriched in pathogenetic (57.1%) and likely pathogenetic (32.3%) mutations, compared to non-pathogenetic (26.2%) (p = 0.026)
Lancheros et al Nutrients (2022)	Case–control study	Spain/Caucasian	204 cases of NSCLC; 408 unmatched controls	NSCLC	Any cancer (unspecified degree and tumors among relatives)	FHC of 49.5% (cases)	13.24% of never smokers, 39.71% of former smokers, 47.96% of current smokers	FHC was associated with diagnosis of NSCLC (OR 15.2, 95%CI: 9.55–25.2)	The Vitamin D Receptor (VDR) BsmI rs1544410-AA germline SNP was associated with lower risk of NSCLC after adjusting for FHC and smoking
Rifkin et al. Clin Lung Cancer (2023)	Case–control study	United Kingdom/Withes (94.7%)	2050 patients with lung cancer/198533 controls	Lung cancer (not specified)	Lung cancer (unspecified degree among relatives)	FH of lung cancer of 21.0%	20% of never smokers, 46.6% of former smokers, 33.4% of current smokers	FH of lung cancer was significantly associated with diagnosis of lung cancer	FH of lung cancer (whole cohort including cases and controls) was significantly associated to germline mutations in 3 significant genes (ATM, BRCA2, TP53)

NSCLC: non-small cell lung cancer; SCLC: small cell lung cancer; OR: odds ratio; 95%CI 95% confidence intervals; SNPs: single nucleotide polymorphisms

### Studies investigating associations between FHC and lung cancer somatic features.

Seven studies reported on the potential association between FHC and lung cancer somatic features (Table 4) [15, 52, 58–62]. Three studies did not confirm significant associations between FHC and somatic microsatellite instability status [58], somatic DDR genes status [52], or *KRAS* mutational status [59], while 2 studies reported a significant association between FHC and* EGFR* mutation [60, 61]. In addition, another study reported an association between FHC and the occurrence of multiple somatic mutations in patients tested for multiple genes [62].

**Table 4:** summary of the included studies reporting on the potential association between FHC and somatic features

Study	Design	Country/race	Study population	Histology	Type of FHC	Prevalence of FHC among cases	Smoking status details (cases)	Main findings	Additional findings
Suzuki et al Br J Cancer (1998)	Observational, retrospective	Japan/Asian	136 female patients with NSCLC with FHC and 243 female patients with NSCLC and no FHC	NSCLC	Any cancer (first-degree relatives)	FHC of 35.9% among the study population	69.4% of never smokers	Microsatellite instability (MSI) was associated to high burden of FHC (at least 3 relatives diagnosed with cancer) compared to controls	No significant difference in clinicopathological characteristics between patients with MSI and those without MSI
Yilmaz et al Int J Environ Res Public Health (2014)	Observational, retrospective	USA/not available	108 patients with NSCLC and 116 with colorectal cancer	NSCLC	Any cancer (unspecified degree and tumors among relatives)	FHC of 63.2% among patients with NSCLC	Among KRAS positive 91.7% of ever smokers Among KRAS negative 69.2% of ever smokers	FHC was not associated with KRAS mutational status	Among KRAS positive, positive FHC was more frequently reported among patients with NSCLC than those with colorectal cancer
Cheng and Cheng J Thorac Dis (2015)	Case–control study (retrospective)	Taiwan/Asian	85 never smoker (< 100 cigarette in lifetime) patients with NSCLC and positive FHC; 161 patients with NSCLC with no FHC (serving as controls)	NSCLC	Any cancer (first-degree and second-degree relatives) Categorization into pulmonary and non-pulmonary tumors	FHC of 34.5% among the whole study population	–	FHC was associated with EGFR mutation status (OR 5.9, 95%CI: 3.3–10.6)	FH of pulmonary cancer was associated with EGFR mutation status (OR 7.5, 95%CI: 3.4–16.3) FH of non-pulmonary cancer was associated with EGFR mutation status (OR 5.0, 95%CI: 2.5–10.0)
Hsu et al Oncotarget (2016)	Observational, retrospective	Taiwan/Asian	131 patients with EGFR positive NSCLC and FH of lung cancer; 1582 patients with EGFR positive NSCLC and no FHC (serving as controls) Additional sub-cohort of patients with EGFR positive NSCLC with at least 2 relatives with lung cancer	Adenocarcinoma (EGFR positive patients)	Lung cancer (first-degree relatives)	FH of lung cancer of 7.6% among the whole population	80.9% of never smokers, 19.1% of ever smokers	FH of lung cancer was associated with EGFR mutational status in multivariable models (OR 1.68, 95%CI: 1.06–2.67)	FH of lung cancer was associated with younger age at diagnosis, with lower TNM stage correlated with younger age at diagnosis No additional findings from cohort 2
Cortellini et al J Hematol Oncol (2022)	Case control study	Italy/not specified	723 patients with NSCLC treated with pembrolizumab (cases); 652 patients with NSCLC treated with chemotherapy (controls)	NSCLC	Any cancer in lineal line (descendants or ascendants) and collateral line (non-descendants/ascendants, relatives)	FHC of 37.5% (cases) 49.3% (controls)	Pembrolizumab cohort: 12.4% of never smokers and 87.6% of ever smokers Chemotherapy cohort: 12.6% of never smokers and 87.4% of ever smokers	High burden of FHC associated with improved outcomes compared to low burden/negative FHC among patients treated with pembrolizumab only	FHC was not associated with tumor mutational burden, PD-L1 status and somatic DNA damage and response genes status
Chang et al Future Oncol (2022)	Observational, retrospective	China/Asian	517 patients with NSCLC categorized into wild-type, single-gene mutation (50.87%) and concomitant mutations (11.99%)	NSCLC	Any cancer (unspecified degree and tumors among relatives)	FHC of 6.18% among the whole cohort	61.3% of never smokers, 15.8% of former smokers, 23.40% of current smokers	FHC was significantly associated with the presence of concomitant mutations, compared to single gene/wild-type group	–
Lashkrizedeh et al Clin Respir J (2023)	Cross-sectional study	Iran/not available	100 patients with lung cancer	NSCLC and SCLC	FH of lung cancer (unspecified degree among relatives)	FH of lung cancer of 5%	16% of never smokers, 84% of ever smokers	No association between FH of lung cancer and HER2/neu status	–

NSCLC: non-small cell lung cancer; SCLC: small cell lung cancer; OR: odds ratio; 95%CI 95% confidence intervals

### Studies investigating associations between FHC and other lung cancer features.

Nine studies included in this subgroup reported on associations between FHC and other lung cancer features (Table 5) [18, 19, 21, 22, 63–67]. One study reported a link between younger age at diagnosis female gender and FHC [63], one study reported an increased prevalence of FH of breast cancer among female patients with lung cancer [64], while another study reported a 10-years increasing trend over time for the prevalence of FHC [22]. Importantly, one study reported a significant association between FHC and smoking [19], while another study reported that FH of lung cancer was more frequent among young women, with synergistic effect with smoking and coil exposure in determining the younger age at diagnosis [21].

**Table 5:** summary of the included studies reporting on the potential association between FHC and other features

Study	Design	Country/Race	Study Population	Histology	Type of FHC	Prevalence of FHC among cases	Smoking status details (cases)	Main findings	Additional findings
Ambrosone et al Cancer (1993)	Observational, retrospective	USA/not available	339 female; 533 male patients with lung cancer	NSCLC	Any cancer (first-degree relatives)	FHC of 44.3% among the study population	9% of never smokers, 91% of ever smokers	Age at diagnosis < 57 years was significantly associated with FHC among patients with squamous cell carcinoma patients (p < 0.01) Female diagnosed at < 57 years reported higher prevalence of positive FHC (75%) compared to male (57%) (p < 0.05)	Reduced prevalence of positive FHC among non-smokers with adenocarcinoma histology than among non-smoker with squamous cell carcinoma histology and SCLC
Tsuchiya et al Jpn J Clin Oncol (2007)	Observational, cross sectional	Japan/Asian	1566 patients with lung cancer	NSCLC and SCLC	Gastric, colon, lung, breast cancer (first-degree relatives)	FH of lung cancer 12% and 14% among male and female cases. FH of other cancers of 29.7% and 33.3% among male and female cases	Among males: 5% of never smokers, 95% of ever smokers. Among females: 68.3% of never smoker, 31.7% of ever smokers	Increased prevalence of FH of breast cancer among female cases compared to male cases	No statistical difference in first-degree FH of stomach, colon, and lung cancer between male and female cases
Isla et al Anticancer Res (2016)	Case–control study	Spain/not available	876 female patients with FHC and 886 female patients without FHC	NSCLC and SCLC	Any cancer (first-degree and second-degree relatives)	FHC of 42.5% among the whole cohort; 43.5% among patients with SCLC; 42.4% among patients with NSCLC	Among patients without FHC: 43% of never smokers, 15.7% of former smokers, 40.5% of current smokers Among patients with FHC: 36.4% of never smokers, 15.6% former smokers, 47.2 current smokers	FHC was not associated with tumor type (NSCLC vs SCLC) Positive smoking personal history was significantly associated with FHC (p = 0.036)	Positive FHC was associated with shorter OS (23 vs 25.3 months, p = 0.029)
Banik et al Health Qual Life Outcomes (2017)	Observational, prospective	Poland/not available	102 patients undergoing resection for NSCLC	NSCLC	Any cancer (first-degree relatives)	FHC of 54%	NA	FHC was not associated with patients’ awareness FHC was significantly associated with lower physical quality of life. FHC was associated with decreased self-efficacy and emotional quality of life	FHC was not associated with with stage at diagnosis/gender Increasing symptoms reported among women with FHC
Chen et al Thorac Cancer (2019)	Observational, retrospective	China/Asian	410 patients with NSCLC screened for tobacco and coal exposure	NSCLC	FH of lung cancer (defined as 3 cases of lung cancer among first-degree relatives)	FH of lung cancer of 46.8%	43.7% of never smokers, 56.3% of ever smokers	FH of lung cancer was more frequent in women and younger age at diagnosis Synergist association between FH of lung cancer, increasing smoking and coal exposure and younger age at diagnosis	FH of lung cancer more frequent among patients with NSCLC, patients with stage IV tumors and among patients with ALK positive NSCLC Synergist association between FH of lung cancer, increasing smoking and coal exposure and ALK positive status, with no association between FH of lung cancer and ALK status among those with low exposure
Gaur et al J Cancer Res Ther (2020)	Case–control study	India	201 patients with lung cancer; 100 unmatched healthy controls	NSCLC, SCLC, and others	Any cancer (unspecified degree and tumors among relatives)	FHC of 5.4%	23.4% of never smokers, 31.8% of former smokers, 41.3% of current smokers	FHC of 5.4%	-
Zang et al Ann Thorac Surg (2020)	Observational, retrospective	China/Asian	7184 patients with primary lung cancer	NSCLC, SCLC and others	Any cancer and lung cancer (unspecified degree among relatives)	FH of any cancer 8.3%, FH of lung cancer 3.2%	Prevalence of ever smokers in the whole cohort was 57.6%	10-years increase in FHC (from 7% to 11.5%, p < 0.001)	FH of lung cancer did not increase over the 10-years study window
Chen et al Front Surg (2022)	Case–control study	Taiwan/Asian	79 non intubated (cases) and 158 intubated (controls) patients after video-assisted thoracoscopic surgery (VATS) resection for lung cancer and aged at least 75 years	NSCLC	Any cancer (unspecified degree and tumors among relatives)	FHC of 11.4% (whole study population)	24.1% of never smokers	FHC was not associated to intubation	–
Li et al Front Oncol (2022)	Observational, retrospective	China/Asian	31 patients with pulmonary mucoepidermoid carcinoma	Mucoepidermoid carcinoma	Any cancer (unspecified degree among relatives)	FH of any cancer 19.9%	51.6% of ever smokers	FH of any cancer 19.9%	No association between FHC and tumor grade

NSCLC: non-small cell lung cancer; SCLC: small cell lung cancer

## FAHIC lung—methods/design

### Study design and objectives

The FAHIC—Lung study (observational, prospective, multicenter study to investigate the family history of cancer in patients with non-small cell lung cancer) is a cross-sectional/prospective, observational, multicenter study. Consecutive patients with histologically diagnosed NSCLC will be enrolled, regardless of their age, TNM stage, smoking status, and other clinicopathologic characteristics. ClinicalTrials.gov identifier: NCT06196424.

The primary objective of the study is the identification of FHC patterns and within-family clusters of other risk factors to address patients with NSCLC for systematic genetic counseling for germline next-generation sequencing (NGS) testing to identify PGVs and likely PGVs. Secondary objectives include the description of clinicopathological and oncological characteristics of patients with NSCLC according to FHC patterns.

Patients’ family history will be carefully collected by investigators through a dedicated self-reported study questionnaire, which has been developed for the purpose of this study and validated by the genetic expert of the steering committee (F.G.) (Supplementary file 2). Study questionnaire will focus on: (1) family history of cancer; (2) type of tumors/primary tumor sites among relatives with history of cancer; (3) age at diagnosis among relatives with history of cancer; (4) biological sex of relatives with history of cancer; (5) exposure to tobacco smoking and smoking habits among relatives with history of cancer; (6) geographical origin of participants and relatives with history of cancer; (7) personal history of multiple malignancies; (8) potential professional and environmental exposure to carcinogens of participants and relatives with history of cancer; (9) ethnicity of both participants and relatives with history of cancer.

**Fig. 2:**
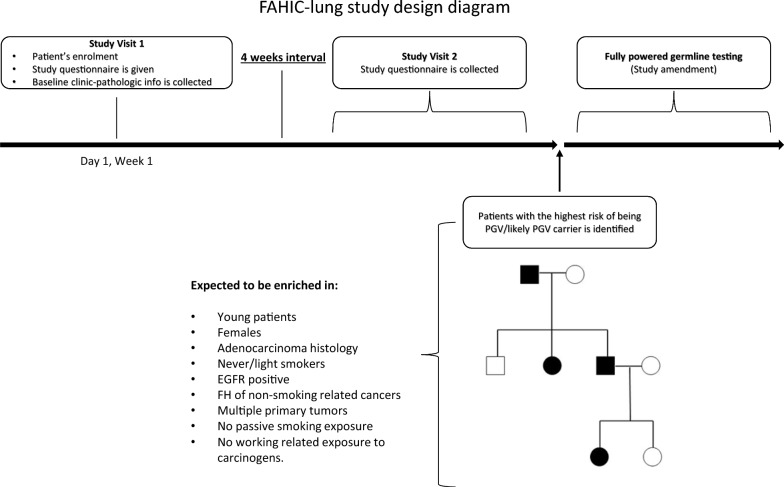
FAHIC-lung study design diagram

To minimize risks of recalling bias, patients will be followed up for four weeks through two study visits: the first study visit at enrolment and the follow-up study visit. During the first study visit all patient’s clinic-pathologic will be collected and study participants will be given the ad-hoc questionnaire, which will be returned to the study personnel at the follow-up study visit (Fig. 2).

The following clinic-pathologic characteristics will be collected: (1) smoking status (active/passive, package/year, total years of smoking); (2) Eastern Cooperative Oncology Group Performance Status (ECOG-PS); (3) age at diagnosis; (4) tumor histology; (5) tumor stage at diagnosis according to the 8th edition of TNM staging system; (6) ethnicity; (7) professional and environmental exposure to carcinogens; (8) programmed death ligand-1 tumor proportion score (PD—L1 TPS); (9) any available oncogenic drivers including *EGFR, KRAS, BRAF, c-MET*, mutations and *ALK, ROS-1, RET, NTRK* translocation/gene fusions; (10) personal history of other synchronous/metachronous primary malignancies.

The study plan includes an observational phase and an analytical phase:

*Observational phase*: after collecting participants’ questionnaires, we will first reconstruct patients’ family trees with additional information on how other potential risk factors, such as smoking history and exposure to professional/environmental carcinogens, segregate within the families with a history of cancer.

*Analytical phase*: once we have identified family clusters of malignancies and risk factors potentially associated with the highest risk of being carriers of germline PGVs or likely PGVs, we will proceed with the collection of blood samples for germline testing in a subgroup of patients. This will enable us to assess and compare the prevalence of PGVs/likely PGVs between patients more likely to be carriers and the control cohort. This approach aims to achieve a robust comparison, minimize systematic referrals to genetic counseling for all NSCLC patients, and optimize NGS testing requests outside the research setting. Considering the validity and comprehensiveness of high-throughput techniques in identifying PGVs/likely PGVs [68], we will assess the germline status of the groups of interest through whole exome sequencing (WES) after DNA extraction from blood samples in a two step analysis.

In the first step, the raw sequencing data (FASTQ files) will undergo bioinformatic processing. Mapping will be performed using a high-throughput aligner to ensure accurate alignment of the sequenced reads to the human genome. Variant calling will then be conducted to identify deviations from the reference genome. Filtering and annotation of these variants will focus on a pre-specified list of pre-specified genes known to be associated potentially associated with cancer (Supplementary file 3). This curated gene list will be used to prioritize PGVs/likely PGVs variants. Online tools will be utilized for variant prioritization, organizing the genes based on their correlation with lung cancer, thus enabling us to pinpoint the most relevant variants for further investigation.

In the second step, we aim to discover novel variants that may contribute to lung cancer predisposition. This phase involves a more exploratory analysis of the FASTQ data, looking beyond the known pathogenic variants. We will leverage the extensive genealogical data we have collected on the patients’ family histories to identify potential new genetic markers. The stored FASTQ files will be re-analyzed to detect previously unreported variants, incorporating bioinformatics tools and techniques for variant discovery. These include advanced algorithms for variant detection and annotation, as well as integrative approaches to assess the potential pathogenicity of novel variants. The integration of genealogical data will enhance our ability to correlate these novel variants with familial patterns of lung cancer, potentially uncovering new genetic predispositions. This comprehensive approach ensures that we maximize the utility of the sequencing data, providing a robust platform for both targeted and discovery-driven genetic analysis.

### Participants selection

Inclusion Criteria include: (1) histopathological diagnosis of NSCLC (all stages); (2) age ≥ 18 years old; (3) signed written informed consent; (4) availability of familiar and/or personal anamnestic data of cancer. Exclusion Criteria include: (1) unavailability of familiar and/or personal anamnestic data of cancer; (2) patient’s refusal.

### Statistical plan and sample size

The sample size of patients enrolled has been determined only for the observational phase of the study. This determination focuses on identifying patients who are more likely to be carriers of pathogenic germline variants (PGVs) or likely PGVs. This approach acknowledges the lack of information on the prevalence of germline PGVs/likely PGVs in patients with NSCLC who are not selected based on family history of cancer (FHC), as well as the limited knowledge regarding the potential characteristics that will define our group of interest. We hypothesized a prevalence of 10% of participants with an especially enriched family history of cancer to be directed to systematic germline testing; assuming a confidence level of 95% with a total width for the confidence interval of 0.1 (precision of ± 5%), the minimum number of subjects needed to properly describe the group of interest, following a binomial “exact” calculation of the sample size, is 175. To account for potential dropouts, we will enroll a minimum of 180 patients.

Descriptive statistics will be used as appropriate to report FHC data, the distribution of within-family other risk factors, and baseline clinicopathologic characteristics. Analyses will be performed using R-Studio software (R Core Team, 2021), and MedCalc® Statistical Software version 20 (MedCalc Software Ltd, Ostend, Belgium; https://www.medcalc.org; 2021).

## Discussion

To the best of our knowledge, this is the first systematic review summarizing the available evidence on the role of FHC in patients with lung cancer, and the FAHIC-lung study (NCT06196424) is the first cross-sectional/prospective study specifically designed to identify patients with NSCLC more likely to be carrier of PGVs/likely PGVs, that should be systematically referred to genetic counselling and germline testing.

Our review shows that few studies have focused on the family history of cancer (FHC) in patients with lung cancer, resulting in overall heterogeneous results, beginning with the extremely wide range of FHC and family history of lung cancer rates. The category with the highest number of reports included studies assessing FHC as a potential risk factor for developing lung cancer. However, even in this category, the results were largely discordant, with a variety of different approaches and categorizations. Most of the included studies followed a retrospective approach, which is inherently associated with recall bias in collecting family history information, and none used questionnaires specifically designed to collect FHC. To mitigate this bias, we developed our ad-hoc study questionnaire, while the cross-sectional/prospective approach with the 4-week interval will allow study participants to gather and report FHC information as carefully as possible.

Something that set lung cancer apart from other malignancies, where the FHC has an established role in defining the probability of being a carrier of PGVs/likely PGVs, such as ovarian, breast, prostate, and colorectal cancer, is the role of smoking. As mentioned, smoking history represents the main risk factor for lung cancer [6], several evidence shows that passive smoking from family members can be a detrimental factor and that even the smoking habit can be “inherited”, with a sort of intergenerational transmission [69, 70]. The FAHIC-lung questionnaire will allow us to mitigate this potential bias as well, collecting smoking habit information and environmental exposure to carcinogens among patients’ relatives with cancer.

More than a half of the studies that assessed FHC and FH of lung cancer as a potential risk factor for lung cancer concluded that FHC plays a detrimental role, with a potential synergistic effect with smoking, that seems even more pronounced among young/female patients. Our systematic review also suggests that younger patients, female, Asian, and never/light smokers may be especially enriched in FHC, although with no clear/conclusive results, while no somatic genomic feature seems to be significantly associated with FHC, except for *EGFR* mutations.

Recently, increasing attention has been focused on the study of germline mutations as risk factor for lung cancer, highlighting how DDR genes alterations can be found among patients with lung adenocarcinoma, even in the context of wider within-family primary tumors spectrums, including breast/pancreatic cancers or hematological malignancies [10]. Even in the context of TP53-associated genetic susceptibility, FHC is gaining a clearer role, to the point of recommending genetic counselling for patients with lung adenocarcinoma younger than 46 years old and with an especially enriched FHC or personal history of multiple primary tumors [71].

Importantly, in our systematic review only one of the studies that investigated the multifaceted role of germline mutations reported a significant enrichment among patients with FHC [46]. Rifkin and colleagues first reported a systematic review on the evidence linking germline mutations with lung cancer risk, then validated through a large case–control study of patients undergoing germline whole exome sequencing (WES) the significant association between lung cancer risk and *ATM*,* BRCA2 *and *TP*53 pathogenetic/likely pathogenetic germline mutations [46]. However, despite the overall enrichment among controls, variant-based and gene-based analyses showed a low prevalence of germline PGV/likely PGV in both cases and controls [46]. In addition, they reported a higher rate of carriers among study participants with FH of lung cancer compared to those without, but with a very low overall prevalence (0.8% vs 0.7% for the combination of *ATM/BRCA2/TP53*) [46], suggesting that a simplified collection of FHC information is not enough to identify patients with the highest probability of being carriers and to properly optimize germ-line NGC access.

Among gene-specific susceptibility for lung cancer, *EGFR*-associated one needs a special mention. Genetic counselling is already recommended for patients with somatic *EGFR* positive NSCLC younger than 50 years, regardless of their family history [10], however, a proper syndromic *EGFR*-associated lung cancer should be suspected in the case of the novo *EGFR* T790M mutations, especially with a somatic variant allele frequency (VAF) ≥ 35% [10, 72], with even more rare *EGFR* variants, such as V834L and V843I being increasingly recognized [73, 74]. Lastly, we will have to consider the complexity related to the multifaceted role of multiple primary tumors. Beyond the consisting evidence linking DDR genes mutations to a personal history of multiple malignancies, recent studies reported on the potential role of pleiotropic loci in determining the risk of multiple malignancies [75].

Our study plan has, however, some limitations. First, we will have to rely on patients' ability and willingness to reconstruct their family history, therefore the recall bias will exert a certain effect despite the cross/sectional prospective approach. In addition, we have no strictly predefined definition of potential family clusters to be analyzed. However, we can anticipate that the identified group of interest will likely include young female patients with adenocarcinoma histology, never or light smokers, patients with *EGFR *mutations, patients with a history of multiple primary tumors, and patients with a high burden of family history. This high burden of family history is particularly expected to be enriched in non-smoking associated cancers, including lung cancer, and in the DDR-genes associated cancer spectrum, such as breast, ovarian, prostate, melanoma, and pancreatic cancers.To ensure a comprehensive analysis, we also plan to incorporate other factors collected through our detailed questionnaire. These factors include smoking habits of the patients, passive smoking exposure, working exposure to carcinogens, and smoking habits of family members. By evaluating these additional factors, we aim to identify within-family clusters of other risk factors. Specifically, we will focus on selecting patients without a history of passive smoking, identifying patients with a younger age at diagnosis among their relatives with cancer, and considering patients with low working exposure to carcinogens. Despite having these anticipations, we have deliberately chosen to adopt an unbiased approach without pre-established features to define patients for germline tests. Considering the very low prevalence of germline mutations reported so far [46], this strategy allows for a more comprehensive and inclusive analysis, ensuring that we do not overlook any potential associations or risk factors to unravel the complexity of FHC information and identify patients especially enriched in PGVs/likely PGVs. Furthermore, considering that this is an observational study, we decided to adopt a two steps approach, in order to identify patients at risk as a first step. This, to minimize the potential clinical implications for study participants and let their treating physicians refer them to genetic counseling as per their existing clinical practice. Once the group of interest will be identified, we will amend the protocol to collect blood samples and allocate fundings for germline testing. Lastly, we have to consider that the FAIHC lung study is being conducted in Italy, therefore the study population will mostly consist of white/Caucasian patients. Although this will prevent us from gathering broader information on the potential implications of different races, we will be able to focus and obtain reliable results on patients with European ancestry.

In the context of a worldwide progressive implementation of chest computed tomography based screening programs in subject with smoking history [76], and considering the initial evidence of the potential benefit of screening programs among never smokers and other subjects potentially enriched in FHC/PGVs [77], identifying patients with the highest risk of being carrier of PGVs/likely PGVs would be extremely important to develop dedicated preventing measures in non-smoker subjects. Considering the costs of commercially available germline NGS tests and the potential preventive, prognostic, and therapeutic implications of the detection of germline mutations related to familial cancers, we believe that establishing FHC patterns to identify a subgroup of patients especially enriched in PGVs to direct to germline screening outside of the research setting, would be extremely helpful in optimizing resources, spare time and eventually improve patients’ outcomes.

### Supplementary Information

Supplementary file 1.

Supplementary file 2.

Supplementary file 3.

Supplementary file 4.

## Data Availability

This systematic review does not involve the generation of new data. The data analyzed in this study are derived from publicly available studies and publications that are cited within the paper. All sources of data, including databases and search strategies used to identify relevant studies, are described in the Methods section. Readers interested in accessing the underlying data can refer to the referenced studies and publications for more detailed information. Due to data management regulations, individual patient-level data from the FAIHC-lung study are not available. However, inquiries from third parties can be directed to the corresponding author.
