# Supplementary material for: Clinical implications of the family history in patients with lung cancer: a systematic review of the literature and a new cross-sectional/prospective study design (FAHIC: lung)
Source: J Transl Med. 2024 Jul 31;22:714. doi: 10.1186/s12967-024-05538-4 (PMC11293007; doi:10.1186/s12967-024-05538-4)
Supplement: Supplementary file 1 [file 12967_2024_5538_MOESM1_ESM.docx]

| **Category** | **Details** |
| --- | --- |
| **FHC as a Risk Factor (27 studies)** | |
| Increased risk for subjects with FHC | More than half of the studies report an increased risk for subjects with FHC |
| Specific associations (e.g., age, gender, smoking status) | Specific associations based on age, gender, and smoking status |
| Varied methodologies and outcomes | Varied methodologies and outcomes across studies |
| **Impact on Clinical Outcomes (5 studies)** | |
| No association in some studies | Some studies found no association between FHC and clinical outcomes |
| Differential effects in others (e.g., improved outcomes with PD-1 immunotherapy) | Other studies report differential effects, e.g., improved outcomes with PD-1 immunotherapy |
| Varied impacts on survival rates | Varied impacts on survival rates |
| **Associations with Germline Mutations (12 studies)** | |
| Mixed results on enrichment of germline mutations | Mixed results on the enrichment of germline mutations |
| Significant effects in certain studies (e.g., ATM, BRCA2, TP53) | Significant effects in certain studies, e.g., ATM, BRCA2, TP53 |
| Synergistic effects with other factors (e.g., XRCC3/XRCC4 variants) | Synergistic effects with other factors, e.g., XRCC3/XRCC4 variants |
| **Associations with Somatic Features (7 studies)** | |
| No significant associations in some studies | Some studies found no significant associations |
| Significant associations in others (e.g., EGFR mutation) | Other studies found significant associations, e.g., with EGFR mutation |
| Varied somatic features assessed | Varied somatic features assessed |
| **Associations with Other Features (9 studies)** | |
| Associations with demographics (e.g., age, gender) | Associations with demographics, e.g., age and gender |
| Increased prevalence of FH of other cancers (e.g., breast cancer) | Increased prevalence of family history of other cancers, e.g., breast cancer |
| Associations with smoking and environmental factors | Associations with smoking and environmental factors |
| Varied other features assessed | Varied other features assessed |

**Synoptic table with organization of results**
