# Supplementary material for: Clinical implications of the family history in patients with lung cancer: a systematic review of the literature and a new cross-sectional/prospective study design (FAHIC: lung)
Source: J Transl Med. 2024 Jul 31;22:714. doi: 10.1186/s12967-024-05538-4 (PMC11293007; doi:10.1186/s12967-024-05538-4)
Supplement: Supplementary file 2 [file 12967_2024_5538_MOESM2_ESM.docx]

**CLINICAL IMPLICATIONS OF FAMILY HISTORY OF CANCER IN PATIENTS AFFECTED BY NON-SMALL CELL LUNG CANCER**

**Patient’s questionnaire**

1. **Patient’s data**

**PERSONAL DATA**

Name: ________________________________

Surname: ______________________________

Date of birth: _________________________

**SMOKING HISTORY**

Current smoker

Never smoker

Former smoker (at least 6 months)

**If smoking history, which kind of cigarettes?**

Conventional

Electronic cigarettes

Both

Pipe/cigar

**Age at smoking starting: __________________**

**Age at smoking cessation: _________________**

**Average cigarettes smoked per day: _______**

**WORK HISTORY**

Kind of job (state “NO” if never worker): ___________________________

Current worker YES NO (tick proper answer)

If current or former worker, state kind of job: __________

If current or former worker, age at starting: __________

If former worker, age at conclusion: __________

**GEOGRAPHICAL DATA**

Where are your parents from? ______________________ (please state city and region)

Last age lived with your parents: __________

Where are you currently living? ______________________ (please state city and region)

Did you live in other cities? (please state in chronological order)

1. : ___________________________ (please state city and region)
2. : ___________________________ (please state city and region)
3. : ___________________________ (please state city and region)
4. : ___________________________ (please state city and region)

**FAMILY HISTORY OF CANCER**

**Has your FATHER ever received oncological diagnosis:**

I have never known him/I cannot provide information

NO

SI

**If yes**

**Which kind of cancer?** (multiple answers allowed)

Lung Cancer

If Lung cancer, whereas possible, tick if:

Non-Small Cell Lung Cancer (Adenocarcinoma, Squamous cell, NOS)

Small Cell Lung Cancer

Unknown/I cannot remember

Other primary thoracic (for example, pleural)

Central nervous system (brain)

Primary skeletal

Liver

Intestinal

Head & neck

Stomach

Breast

Pancreas

Melanoma/Skin

Prostate

Others

**Father’s age at diagnosis**

Punctual age, if known: _______

Younger than 50 years old:

Between 50 and 70 years old:

Older than 70 years old:

**Has your father ever smoked:**

YES NO I don’t know (tick proper answer)

**Has your MOTHER ever received oncological diagnosis:**

I have never known him/I cannot provide information

NO

SI

**If yes**

**Which kind of cancer?** (multiple answers allowed)

Lung Cancer

If Lung cancer, whereas possible, tick if:

Non-Small Cell Lung Cancer (Adenocarcinoma, Squamous cell, NOS)

Small Cell Lung Cancer

Unknown/I cannot remember

Other primary thoracic (for example, pleural)

Central nervous system (brain)

Primary skeletal

Liver

Intestinal

Head & neck

Stomach

Breast

Pancreas

Melanoma/Skin

Ovarian

Uterine

Others

**Mother’s age at diagnosis**

Punctual age, if known: _______

Younger than 50 years old:

Between 50 and 70 years old:

Older than 70 years old:

**Has your mother ever smoked:**

YES NO I don’t know (tick proper answer)

**Do you have brothers/sisters?**

YES NO I don’t know (tick proper answer)

**PLEASE PROVIDE DATA IF YOU HAVE AT LEAST ONE SIBLING**

**If you have AT LEAST ONE BROTHER OR SISTER, PLEASE STATE:**

Number of brothers: __________

**Number of brothers with oncological diagnosis: ________**

Number of sisters: __________

**Number of sisters with oncological diagnosis: ________**

**BROTHER 1**

**Which kind of cancer?** (multiple answers allowed)

Lung Cancer

If Lung cancer, whereas possible, tick if:

Non-Small Cell Lung Cancer (Adenocarcinoma, Squamous cell, NOS)

Small Cell Lung Cancer

Unknown/I cannot remember

Other primary thoracic (for example, pleural)

Central nervous system (brain)

Primary skeletal

Liver

Intestinal

Head & neck

Stomach

Breast

Pancreas

Melanoma/Skin

Prostate

Others

**Brother’s age at diagnosis**

Punctual age, if known: _______

Younger than 50 years old:

Between 50 and 70 years old:

Older than 70 years old:

**Has your brother ever smoked:**

YES NO I don’t know (tick proper answer)

**BROTHER 2**

**Which kind of cancer?** (multiple answers allowed)

Lung Cancer

If Lung cancer, whereas possible, tick if:

Non-Small Cell Lung Cancer (Adenocarcinoma, Squamous cell, NOS)

Small Cell Lung Cancer

Unknown/I cannot remember

Other primary thoracic (for example, pleural)

Central nervous system (brain)

Primary skeletal

Liver

Intestinal

Head & neck

Stomach

Breast

Pancreas

Melanoma/Skin

Prostate

Others

**Brother’s age at diagnosis**

Punctual age, if known: _______

Younger than 50 years old:

Between 50 and 70 years old:

Older than 70 years old:

**Has your brother ever smoked:**

YES NO I don’t know (tick proper answer)

**BROTHER 3**

**Which kind of cancer?** (multiple answers allowed)

Lung Cancer

If Lung cancer, whereas possible, tick if:

Non-Small Cell Lung Cancer (Adenocarcinoma, Squamous cell, NOS)

Small Cell Lung Cancer

Unknown/I cannot remember

Other primary thoracic (for example, pleural)

Central nervous system (brain)

Primary skeletal

Liver

Intestinal

Head & neck

Stomach

Breast

Pancreas

Melanoma/Skin

Prostate

Others

**Brother’s age at diagnosis**

Punctual age, if known: _______

Younger than 50 years old:

Between 50 and 70 years old:

Older than 70 years old:

**Has your brother ever smoked:**

YES NO I don’t know (tick proper answer)

**BROTHER 4**

**Which kind of cancer?** (multiple answers allowed)

Lung Cancer

If Lung cancer, whereas possible, tick if:

Non-Small Cell Lung Cancer (Adenocarcinoma, Squamous cell, NOS)

Small Cell Lung Cancer

Unknown/I cannot remember

Other primary thoracic (for example, pleural)

Central nervous system (brain)

Primary skeletal

Liver

Intestinal

Head & neck

Stomach

Breast

Pancreas

Melanoma/Skin

Prostate

Others

**Brother’s age at diagnosis**

Punctual age, if known: _______

Younger than 50 years old:

Between 50 and 70 years old:

Older than 70 years old:

**Has your brother ever smoked:**

YES NO I don’t know (tick proper answer)

**BROTHER 5**

**Which kind of cancer?** (multiple answers allowed)

Lung Cancer

If Lung cancer, whereas possible, tick if:

Non-Small Cell Lung Cancer (Adenocarcinoma, Squamous cell, NOS)

Small Cell Lung Cancer

Unknown/I cannot remember

Other primary thoracic (for example, pleural)

Central nervous system (brain)

Primary skeletal

Liver

Intestinal

Head & neck

Stomach

Breast

Pancreas

Melanoma/Skin

Prostate

Others

**Brother’s age at diagnosis**

Punctual age, if known: _______

Younger than 50 years old:

Between 50 and 70 years old:

Older than 70 years old:

**Has your brother ever smoked:**

YES NO I don’t know (tick proper answer)

**SISTER 1**

**Which kind of cancer?** (multiple answers allowed)

Lung Cancer

If Lung cancer, whereas possible, tick if:

Non-Small Cell Lung Cancer (Adenocarcinoma, Squamous cell, NOS)

Small Cell Lung Cancer

Unknown/I cannot remember

Other primary thoracic (for example, pleural)

Central nervous system (brain)

Primary skeletal

Liver

Intestinal

Head & neck

Stomach

Breast

Pancreas

Melanoma/Skin

Ovarian

Uterine

Others

**Sister’s age at diagnosis**

Punctual age, if known: _______

Younger than 50 years old:

Between 50 and 70 years old:

Older than 70 years old:

**Has your sister ever smoked:**

YES NO I don’t know (tick proper answer)

**SISTER 2**

**Which kind of cancer?** (multiple answers allowed)

Lung Cancer

If Lung cancer, whereas possible, tick if:

Non-Small Cell Lung Cancer (Adenocarcinoma, Squamous cell, NOS)

Small Cell Lung Cancer

Unknown/I cannot remember

Other primary thoracic (for example, pleural)

Central nervous system (brain)

Primary skeletal

Liver

Intestinal

Head & neck

Stomach

Breast

Pancreas

Melanoma/Skin

Ovarian

Uterine

Others

**Sister’s age at diagnosis**

Punctual age, if known: _______

Younger than 50 years old:

Between 50 and 70 years old:

Older than 70 years old:

**Has your sister ever smoked:**

YES NO I don’t know (tick proper answer)

**SISTER 3**

**Which kind of cancer?** (multiple answers allowed)

Lung Cancer

If Lung cancer, whereas possible, tick if:

Non-Small Cell Lung Cancer (Adenocarcinoma, Squamous cell, NOS)

Small Cell Lung Cancer

Unknown/I cannot remember

Other primary thoracic (for example, pleural)

Central nervous system (brain)

Primary skeletal

Liver

Intestinal

Head & neck

Stomach

Breast

Pancreas

Melanoma/Skin

Ovarian

Uterine

Others

**Sister’s age at diagnosis**

Punctual age, if known: _______

Younger than 50 years old:

Between 50 and 70 years old:

Older than 70 years old:

**Has your sister ever smoked:**

YES NO I don’t know (tick proper answer)

**SISTER 4**

**Which kind of cancer?** (multiple answers allowed)

Lung Cancer

If Lung cancer, whereas possible, tick if:

Non-Small Cell Lung Cancer (Adenocarcinoma, Squamous cell, NOS)

Small Cell Lung Cancer

Unknown/I cannot remember

Other primary thoracic (for example, pleural)

Central nervous system (brain)

Primary skeletal

Liver

Intestinal

Head & neck

Stomach

Breast

Pancreas

Melanoma/Skin

Ovarian

Uterine

Others

**Sister’s age at diagnosis**

Punctual age, if known: _______

Younger than 50 years old:

Between 50 and 70 years old:

Older than 70 years old:

**Has your sister ever smoked:**

YES NO I don’t know (tick proper answer)

**SISTER 5**

**Which kind of cancer?** (multiple answers allowed)

Lung Cancer

If Lung cancer, whereas possible, tick if:

Non-Small Cell Lung Cancer (Adenocarcinoma, Squamous cell, NOS)

Small Cell Lung Cancer

Unknown/I cannot remember

Other primary thoracic (for example, pleural)

Central nervous system (brain)

Primary skeletal

Liver

Intestinal

Head & neck

Stomach

Breast

Pancreas

Melanoma/Skin

Ovarian

Uterine

Others

**Sister’s age at diagnosis**

Punctual age, if known: _______

Younger than 50 years old:

Between 50 and 70 years old:

Older than 70 years old:

**Has your sister ever smoked:**

YES NO I don’t know (tick proper answer)

**ONLY FOR PATIENTS WITH CHILDREN**

**If you have any CHILD, please state:**

Number of sons (MALE): __________

**Number of sons with oncological diagnosis:** __________

Number of daughters (FEMALE): __________

**Number of daughters** **with oncological diagnosis:** __________

**SON 1**

**Which kind of cancer?** (multiple answers allowed)

Lung Cancer

If Lung cancer, whereas possible, tick if:

Non-Small Cell Lung Cancer (Adenocarcinoma, Squamous cell, NOS)

Small Cell Lung Cancer

Unknown/I cannot remember

Other primary thoracic (for example, pleural)

Central nervous system (brain)

Primary skeletal

Liver

Intestinal

Head & neck

Stomach

Breast

Pancreas

Melanoma/Skin

Ovarian

Uterine

Others

**Sister’s age at diagnosis**

Punctual age, if known: _______

Younger than 50 years old:

Between 50 and 70 years old:

Older than 70 years old:

**Has your sister ever smoked:**

YES NO I don’t know (tick proper answer)

**DAUGHTER 1**

**Which kind of cancer?** (multiple answers allowed)

Lung Cancer

If Lung cancer, whereas possible, tick if:

Non-Small Cell Lung Cancer (Adenocarcinoma, Squamous cell, NOS)

Small Cell Lung Cancer

Unknown/I cannot remember

Other primary thoracic (for example, pleural)

Central nervous system (brain)

Primary skeletal

Liver

Intestinal

Head & neck

Stomach

Breast

Pancreas

Melanoma/Skin

Ovarian

Uterine

Others

**Sister’s age at diagnosis**

Punctual age, if known: _______

Younger than 50 years old:

Between 50 and 70 years old:

Older than 70 years old:

**Has your sister ever smoked:**

YES NO I don’t know (tick proper answer)

**PLEASE PROVIDE ANY AVAILABLE INFORMATION REGARDING OTHER FAMILY MEMBERS**

**PATERNAL UNCLES (FATHER’S SIBLINGS)**

NUMBER OF FATHER’S BROTHERS AND SISTERS: __________

How many PATERNAL UNCLES received oncological diagnosis (whereas possible, please state degree of kinship, histological type and age at diagnosis)?:

________________________________________________________________________________________________________________________________________________________________________________________________________________________________________________________________________________________________________________________________

**PATERNAL COUSINS (SONS OF FATHER’S SIBLINGS)**

NUMBER OF PATERNAL COUSINS: __________

How many PATERNAL COUSINS received oncological diagnosis (whereas possible, please state degree of kinship, histological type and age at diagnosis)?:

________________________________________________________________________________________________________________________________________________________________________________________________________________________________________________________________________________________________________________________________

**MATERNAL UNCLES (MOTHER’S SIBLINGS)**

NUMBER OF MOTHER’S BROTHERS AND SISTERS: __________

How many MATERNAL UNCLES received oncological diagnosis (whereas possible, please state degree of kinship, histological type and age at diagnosis)?:

________________________________________________________________________________________________________________________________________________________________________________________________________________________________________________________________________________________________________________________________

**MATERNAL COUSINS (SONS OF MOTHER’S SIBLINGS)**

NUMBER OF MATERNAL COUSINS: __________

How many MATERNAL COUSINS received oncological diagnosis (whereas possible, please state degree of kinship, histological type and age at diagnosis)?:

________________________________________________________________________________________________________________________________________________________________________________________________________________________________________________________________________________________________________________________________
