# Supplementary material for: Clinical implications of the family history in patients with lung cancer: a systematic review of the literature and a new cross-sectional/prospective study design (FAHIC: lung)
Source: J Transl Med. 2024 Jul 31;22:714. doi: 10.1186/s12967-024-05538-4 (PMC11293007; doi:10.1186/s12967-024-05538-4)
Supplement: Supplementary file 3 [file 12967_2024_5538_MOESM3_ESM.docx]

**Pre-specified list of genes potentially associated cancer for the initial analysis**

Base substitutions, insertion/deletions and copy number alterations

*ABL1, ACVR1B, AKT1, AKT2, AKT3, ALOX12B, ATRX, AMER1 (FAM123B), APC, AR, ARAF, ARFRP1, ARID1A, ASXL1, ATM, ATR, AURKA, AURKB, AXIN1, AXL, BAP1, BARD1, BCL2L1, BCL2L2, BCL6, BCOR, BCORL1, BRAF, BRD4, BRIP1, BTG1, BTG2, BTK, C11orf30 (EMSY), C17orf39 (GID4), CALR, CARD11, CASP8, CBFB, CBL, CCND1, CCND2, CCND3, CCNE1, CD22, CD274 (PD-L1), CD70, CD79A, CD79B, CDC73, CDH1, CDK12, CDK4, CDK6, CDK8, CDKN1A, CDKN1B, CDKN2A, CDKN2B, CDKN2C, CEBPA, CHEK1, CHEK2, CIC, CREBBP, CRKL, CSF1R, CSF3R, CTCF, CTNNA1, CTNNB1, CUL3, CUL4A, CXCR4, CYP17A1, DAXX, DDR1, DDR2, DIS3, DNMT3A, DOT1L, EED, EGFR, EP300, EPHA3, EPHB1, EPHB4, ERBB2, ERBB3, ERBB4, ERCC4, ERG, ERRFI1, ESR1, EZH2, FAM46C, FANCA, FANCC, FANCG, FANCL, FAS, FBXW7, FGF10, FGF12, FGF14, FGF19, FGF23, FGF3, FGF4, FGF6, FGFR1, FGFR2, FGFR3, FGFR4, FH, FLCN, FLT1, FLT3, FOXL2, FUBP1, GABRA6, GATA3, GATA4, GATA6, GNA11, GNA13, GNAQ, GNAS, GRM3, GSK3B, H3F3A, HDAC1, HGF, HNF1A, HRAS, HSD3B1, ID3, IDH1, IDH2, IGF1R, IKBKE, IKZF1, INPP4B, IRF2, IRF4, IRS2, JAK1, JAK2, JAK3, JUN, KDM5A, KDM5C, KDM6A, KDR, KEAP1, KEL, KLHL6, KMT2A (MLL), KMT2D (MLL2), KRAS, LTK, LYN, MAF, MAP2K1 (MEK1), MAP2K2 (MEK2), MAP2K4, MAP3K1, MAP3K13, MAPK1, MCL1, MDM2, MDM4, MED12, MEF2B, MEN1, MERTK, MET, MITF, MKNK1, MLH1, MPL, MRE11A, MSH2, MSH3, MSH6, MST1R, MTAP, MTOR, MUTYH, MYC, MYCL (MYCL1), MYCN, MYD88, NBN, NF1, NF2, NFE2L2, NFKBIA, NKX2-1, NOTCH1, NOTCH2, NOTCH3, NPM1, NRAS, NT5C2, NTRK1, NTRK2, NTRK3, P2RY8, PALB2, PARK2, PARP1, PARP2, PARP3, PAX5, PBRM1, PDCD1 (PD1), PDCD1LG2 (PD-L2), PDGFRA, PDGFRB, PDK1, PIK3C2B, PIK3C2G, PIK3CA, PIK3CB, PIK3R1, PIM1, PMS2, POLD1, POLE, PPARG, PPP2R1A, PPP2R2A, PRDM1, PRKAR1A, PRKCI, PTCH1, PTEN, PTPN11, PTPRO, QKI, RAC1, RAD21, RAD51, RAD51B, RAD51C, RAD51D, RAD52, RAD54L, RAF1, RARA, RB1, RBM10, REL, RET, RICTOR, RNF43, ROS1, RPTOR, SDHA, SDHB, SDHC, SDHD, SETD2, SF3B1, SGK1, SMAD2, SMAD4, SMARCA4, SMARCB1, SMO, SNCAIP, SOCS1, SOX2, SOX9, SPEN, SPOP, SRC, STAG2, STAT3, STK11, SUFU, SYK, TBX3, TEK, TET2, TGFBR2, TIPARP, TNFAIP3, TNFRSF14, TP53, TSC1, TSC2, TYRO3, U2AF1, VEGFA, VHL, WHSC1, WHSC1L1, WT1, XPO1, XRCC2, ZNF217, ZNF703, AXIN2, BMPR1A, CHEK2, DICER1, EPCAM, MAX, LZTR1, SPRED1, SDHAF2, NTHL1, SLX4, GALNT12, GATA2, GPC3, GREM1, HOXB13, KIF1B, NSD1, ACD, TERF21P, TERF2IP, TMEM127, FAM175A, RUNX1, RECQL4, RHBDF2, RINT1, PHOX2B, CASR, CDC73, XPA, XPC, DDB2, SPINK1, SMARCE1, CTRC.*

Gene Rearrangements and Translocations

*ALK, BCL2, BCR, BRAF, BRCA1, BRCA2, CD74, EGFR, ETV4, ETV5, ETV6, EWSR1, EZR, FGFR1, FGFR2, FGFR3, KIT, KMT2A (MLL), MSH2, MYB, MYC, NOTCH2, NTRK1, NTRK2, NUTM1, PDGFRA, RAF1, RARA, RET, ROS1, RSPO2, SDC4, SLC34A2, TERC, TERT, TMPRSS2.*
